# Retraction: Shen et al. Investigation of the Ordered Structure in Partially Melted Isotactic Polypropylene. *Polymers* 2021, *13*, 3354

**DOI:** 10.3390/polym15061432

**Published:** 2023-03-14

**Authors:** Junfang Shen, Derong Zhu, Junchao An, Zhiyu Min, Jingbo Chen

**Affiliations:** 1 School of Materials Science and Engineering, Luoyang Institute of Science and Technology, Luoyang 471023, China; shen8009@126.com (J.S.); ajc_1979@163.com (J.A.); 2 Henan Intelligent Manufacturing Engineering Technology Research Center for Building Profile, Luoyang 471023, China; 3 School of Intelligent Manufacturing, Luoyang Institute of Science and Technology, Luoyang 471023, China; 4 School of Materials Science and Engineering, Zhengzhou University, Zhengzhou 450001, China; chenjb@zzu.edu.cn

The published article [1] has been retracted at the request of the authors.

Following its publication, the authors contacted the Editorial Office regarding legal issues related to confidentiality and a copyright issue. Zhengzhou University was not aware that the article had been submitted for publication and did not agree to the publication of the paper in its current form. Thus, in agreement with the *Polymers* Editorial Office and the authors, the paper has been retracted and will be marked accordingly. https://www.mdpi.com/2073-4360/13/19/3354.

This retraction has been approved by the Editor-in-Chief of the journal.

The authors agree to this retraction.
